# PP57 Outcomes Model For Assessing Strategies Improving In Vitro Fertilization Birth Rates

**DOI:** 10.1017/S0266462324002289

**Published:** 2025-01-07

**Authors:** Suzanna Mongan, Juntao Lyu, Hansoo Kim

## Abstract

**Introduction:**

Infertility affects one-sixth of women worldwide, with over seven million assisted reproductive cycles performed annually. Oral dydrogesterone is recommended alternatively for luteal phase support in in vitro fertilization (IVF), preventing miscarriages and improving live birth rates. This study aims to develop an outcomes model comparing oral dydrogesterone treatment with the standard of care in the IVF cycle over a 10-year period.

**Methods:**

A two-level Markov cohort model in Microsoft Excel includes six health states: IVF, pregnancy, miscarriage, live birth, perinatal death, and maternal death. Miscarriage, live birth, and perinatal death are sub-states of pregnancy. Transition probabilities are based on published rates with medical intervention limited to the first 12 weeks of gestation. A sensitivity analysis of treatment was performed. Data from a published meta-analysis of nine dydrogesterone studies for IVF luteal phase support were used. The baseline cohort is 10,000 Australian females undergoing IVF annually over a 10-year period.

**Results:**

Over the 10-year time horizon, compared to standard care, the group treated with dydrogesterone was estimated to increase the number of live births by 3.5 percent (range: 3.4 to 3.7%), reduce the number of miscarriages by 69.4 percent (range: 66.2 to 72.7%), reduce the perinatal death by 10.9 percent (range: 10.4 to 11.4%), reduce the IVF cycles by 11.56 percent (range: 11.0 to 12.11%), and reduce the death of the mothers by 10.9 percent (range: 10.4 to 11.4%).

**Conclusions:**

The outcomes model projected that treatment with oral dydrogesterone significantly reduced the number of miscarriages and improved the number of live births compared to the standard of care used for IVF patients in Australia.

